# Derivation of ligands for the complement C3a receptor from the C-terminus of C5a

**DOI:** 10.1016/j.ejphar.2014.10.041

**Published:** 2014-12-15

**Authors:** Reena Halai, Meghan L Bellows-Peterson, Will Branchett, James Smadbeck, Chris A Kieslich, Daniel E Croker, Matthew A Cooper, Dimitrios Morikis, Trent M Woodruff, Christodoulos A Floudas, Peter N Monk

**Affiliations:** a Institute for Molecular Bioscience, The University of Queensland, Brisbane, QLD 4072, Australia; b Department of Chemical and Biological Engineering, Princeton University, Princeton, NJ, USA; c Department of Infection and Immunity, Sheffield University Medical School, Sheffield, UK; d Department of Bioengineering, University of California, Riverside, CA, USA; e School of Biomedical Sciences, The University of Queensland, Brisbane, QLD 4072, Australia

**Keywords:** C3a receptor, C5a_1_ receptor, Peptide design, *In silico* sequence selection, Computational optimization, Label-free screening

## Abstract

The complement cascade is a highly sophisticated network of proteins that are well regulated and directed in response to invading pathogens or tissue injury. Complement C3a and C5a are key mediators produced by this cascade, and their dysregulation has been linked to a plethora of inflammatory and autoimmune diseases. Consequently, this has stimulated interest in the development of ligands for the receptors for these complement peptides, C3a receptor, and C5a_1_ (C5aR/CD88). In this study we used computational methods to design novel C5a_1_ receptor ligands. However, functional screening in human monocyte-derived macrophages using the xCELLigence label-free platform demonstrated altered specificity of our ligands. No agonist/antagonist activity was observed at C5a_1_, but we instead saw that the ligands were able to partially agonize the closely related complement receptor C3a receptor. This was verified in the presence of C3a receptor antagonist SB 290157 and in a stable cell line expressing either C5a_1_ or C3a receptor alone. C3a agonism has been suggested to be a potential treatment of acute neutrophil-driven traumatic pathologies, and may have great potential as a therapeutic avenue in this arena.

## 1 Introduction

Complement activation proceeds through cascades of enzymatic reactions leading to inflammation, phagocytosis, lysis, and augmentation of antibody production (Markiewski and Lambris, 2007). Two major products of complement activation are the protein fragments C3a and C5a, beneficial in infections but also mediating inflammatory diseases (Peng et al., 2009).

C5a exerts a number of effects through its classical receptor, C5a_1_ (C5aR/CD88) (Klos et al., 2013), such as recruiting neutrophils and macrophages to sites of injury, releasing granule-associated enzymes and vasoactive mediators, increasing vascular permeability and adhesion, inducing smooth muscle contractions and stimulating the release of proinflammatory cytokines. C5a also interacts with a second receptor, C5a_2_, although the functions of this receptor are not fully determined (Croker et al., 2013, Li et al., 2013). Increased amounts of C5a are associated with a number of pathological conditions, including lupus, ischemia/reperfusion injury, Crohn׳s disease, cystic fibrosis, gingivitis, atherosclerosis, myocardial infarction, fibrosis, allergy, diabetes type I, and disorders of the central nervous system (Manthey et al., 2009).

Inhibition of C3a and C5a interactions with their respective receptors, C3a receptor and C5a_1_, has been targeted for drug design (Bellows-Peterson et al., 2012, Monk et al., 2007). However, a recent study has suggested that C3a and C5a_1_ have opposing roles in neutrophil-mediated pathology (Wu et al., 2013). In a model of intestinal injury, the authors of this study showed that C3a receptor is not chemotactic for neutrophils, but rather, constrains neutrophil mobilization. Therefore C3a receptor agonism and C5a_1_ antagonism may have similar therapeutic effects in acute neutrophil-driven pathologies (Schofield et al., 2013).

C3a and C5a are 77- and 74-residue proteins, with 32% sequence identity and similar three-dimensional structures (Klos et al., 2013). A common mechanism of function utilizes the C-terminal cationic domain which inserts into the activation site of respective receptors. Removal of the conserved C-terminal arginine has complex effects on functionality (Croker et al., 2013, Reis et al., 2012). Past drug design efforts have focused on designing peptides and peptidomimetics by modifying the C-terminal regions (Klos et al., 2013). Additional recent efforts have focused on using both sequence and structural templates with innovative computational methods to design C3a receptor agonist and antagonist peptides (Bellows-Peterson et al., 2012), including the current study.

A two-stage de novo protein design framework previously described (Bellows-Peterson et al., 2012, Bellows et al., 2010a, Bellows et al., 2010b, Fung et al., 2008, Fung et al., 2005, Fung et al., 2007, Klepeis et al., 2003, Klepeis et al., 2004) was applied to the design of C5a-derived peptides. The peptides were functionally screened on the reporter cell line, RBL-2H3 transfected with human C3a receptor or C5a_1_ or on human monocyte-derived macrophages (HMDM) using label-free methods that provided cell activation read-outs. Several hits were identified and, based on the activation profiles, these hits appeared to be C3a agonists. Screening of the peptides in the presence of C3a receptor and C5a_1_ antagonists confirmed C3a receptor as the target. Thus modification to the C-terminal sequence conferred C3a agonist activity on C5a peptides.

## 2 Materials and methods

### 2.1 Computational design of peptides

The computational design framework (described in detail in Supplementary material) was broadly as described previously (Bellows-Peterson et al., 2012).

### 2.2 Peptide synthesis

All octa-, hepta-, and hexapeptides tested were synthesized by GenScript (Piscataway, NJ) with acetylated N-termini at >95% purity. Purity was confirmed with HPLC. The C-terminus was unblocked in all peptides.

### 2.3 Cell culture

Human monocytes were isolated from blood donations to the Australian Red Cross Blood Service (Kelvin Grove, Queensland). The isolation of mononuclear cells was as described previously by Halai and Cooper (2012). Briefly, the mononuclear cell layer was separated using density gradient centrifugation with Ficoll-Paque Plus (GE Healthcare). MACS magnetic beads (Miltenyi Biotec) were incubated with the cells for 15 min at 4 °C before passing through an LS column (Miltenyi Biotec) to select for the CD14+ cells. Selected monocytes were plated at a density of 1.5×10^7^ in Iscove׳s Modified Dulbecco׳s Medium (IMDM) (Invitrogen Life Technologies) containing l-glutamine supplemented with 10% Fetal Bovine Serum (FBS), 50 IU/ml penicillin and 50 μg/ml streptomycin. Human macrophage colony-stimulating factor (M-CSF) (Peprotech) was added to media to allow cells to differentiate into human monocyte-derived macrophages (HMDM). Over the 7 day differentiation process, cells were incubated at 37 °C and 5% CO_2_.

### 2.4 Cell impedance assay (xCELLigence RTCA)

HMDM were seeded overnight at 30,000 cells/well in 384-well E-Plates (Roche). Approximately 24 h later, HMDM media were exchanged for serum-free IMDM and equilibrated at 37 °C and 5% CO_2_ for 2 h prior to ligand addition. Ligands were prepared at a final DMSO concentration of 0.5% (or less) in serum-free IMDM. Antagonists PMX53 and SB 290157 were incubated for 1 h prior to agonist addition, at concentration ranges known to cause substantial inhibition of receptor activation in these cells. After agonist addition, measurements were taken continuously for ~1.5 h at 37 °C. The RTCA software was used for data analysis, with statistical analysis using GraphPad Prism 5.0c (GraphPAD Software Inc., San Diego, CA).

### 2.5 Rat basophilic leukemia cell degranulation assay

RBL-2H3 cells transfected with either human C5a_1_ or human C3a receptor (Cain and Monk, 2002) were routinely cultured in Dulbecco׳s Modified Eagle׳s Medium, 10% (v/v) fetal calf serum, and 400 mg/l G-418 at 37 °C in 5% CO_2_. Degranulation was measured by assaying for β-hexosaminidase activity in the cell supernatant, as described previously (Monk et al., 1994). In agonist assays, degranulation was calculated as a percentage of maximal activity in response to 200 nM recombinant human C3a, human C5a (Cain and Monk, 2002), or 10 nM agonist hexapeptide, FLPLAR (Scully et al., 2010). For antagonist assays, cells were pretreated with peptides for 10 min prior to the addition of C3a, C5a, or FLPLAR. EC_50_, IC_50_, and standard error values were obtained by iterative curve fitting in GraphPad Prism v5.0.

## 3 Results

### 3.1 Computational results

For each run (Supplementary Table 1), 200 peptide sequences were generated using the Distance Bin Model (Fung et al., 2008, Rajgaria et al., 2008). This produced 6400 total sequences. Each sequence was subjected to fold specificity calculations using both AMBER and FAMBE-pH and ranked accordingly. Top sequences from each run are combined in Supplementary Table 2. The sequences shown were either the top few sequences in the run, or sequences that showed promising features such as a charged residue in positions 67 or 68 or a hydrophobic residue in position 70 or 72 (as in the native sequence).

The designed peptide sequences displayed a number of patterns, including the mutation to Trp in position 73 (also seen in the most potent antagonists thus far (Finch et al., 1999)) and the preference for a charged residue in position 68. Position 68 showed a preference for a charge residue, either positive or negative. When positively charged, the amino acid of choice was Arg, as opposed to the native Lys. Nearly all the runs tended to rank a hydrophobic residue in position 70 highly, despite the fact that all the templates also allowed hydrophilic residues in that position.

### 3.2 Screening peptides on HMDM using the xCELLigence

**Fig. 1:**
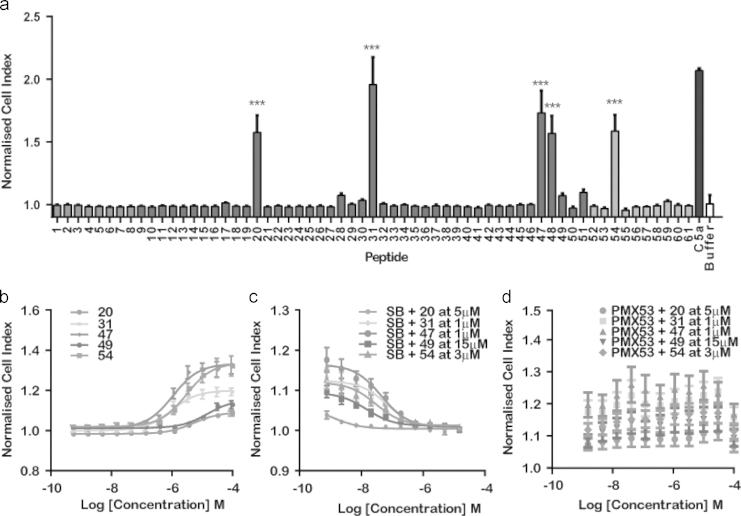
Identification of hits and target on HMDM using the xCELLigence (a) initial screen of 61 peptides at 8 μM on HMDM (*n*=4–6). Asterisk indicates *P*<0.001. Bars are shaded according to peptide length (see Supplementary Table 2), (b) dose–response curves for peptides on HMDM (*n*=3–5), (c) dose-dependent antagonism of response using C3a receptor antagonist SB 290157 (SB) when agonized with the EC_50_ concentrations of hits (*n*=3–5), and (d) dose-dependent antagonism of response using C5a_1_ antagonist PMX53 when agonized with the EC_50_ concentrations of hits (*n*=3–5).

The 61 peptides, initially dissolved in dimethyl sulfoxide (DMSO) at 10 mM, were screened at a single dose of 8 μM on HMDM using an impedance based label-free system, the xCELLigence; DMSO at <0.5% was found to have no effects in this assay. Fig. 1A shows the identification of 5 significant hits (*P*<0.001), peptides 20, 31, 47, 48 and 54. Peptide 49 was identified as a potential hit from independent data (data not shown) despite not evoking a response in the initial screen and so was retested at the full dose range along with the five identified hits (see Fig. 1B). Interestingly, a full dose response could not be obtained for peptide 48 (data not shown). However, peptide 49 showed weak activity, despite failing in the initial single dose screen. The EC_50_ of the hits has been summarized in Table 1.

**Table 1:** Peptides, sequences EC_50_ and IC_50_ concentrations (SB 290157) determined for the five hits, 20, 31, 47, 49 and 54 identified in HMDM, using the impedance-based biosensor, the xCELLigence. The bold-faced letters denote the conserved residues amongst the sequences.

**Peptide number**	**Sequence**	**EC**_**50**_**(M)**	**IC**_**50**_**(M)**
20	Ac-NN**Y**N**LWR**	5.8×10^−6^	1.3×10^−9^
31	Ac-RH**Y**P **LWR**	1.1×10^−6^	6.7×10^−8^
47	Ac-RL**Y**P **LWR**	1.2×10^−6^	4.4×10^−8^
49	Ac-TI **Y**R **LWR**	1.7×10^−5^	1.5×10^−8^
54	Ac-R **Y**P **LWR**	3×10^−6^	2.9×10^−8^

### 3.3 Identifying the target receptor for the hits

**Fig. 2:**
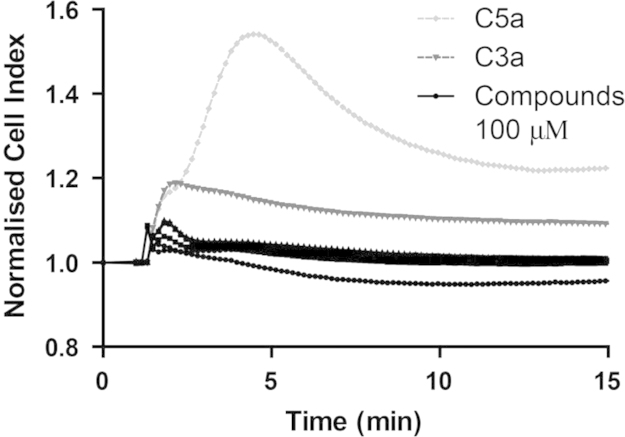
xCELLigence response profiles for C3a and C5a at 100 nM, and peptides 20, 31, 47, 49 and 54 at 100 μM. A two-peak profile is evident for C5a, whereas a single peak profile is apparent for C3a. Peptide profiles more closely resemble C3a activation than C5a activation.

Using the xCELLigence, the hits were tested in the presence of both the C3a receptor and C5a_1_ antagonists, SB 290157 and PMX53, respectively (see Fig. 1C and D). A dose dependent inhibition of the cell index was observed for peptides 20, 31, 47, 49 and 54 in the presence of the competitive antagonist SB 290157 when activated with a concentration of peptide approximating to the EC_50_. The IC_50_ of SB 290157 in the presence of peptides 20, 31, 47, 49 and 54 is highlighted in Table 1. No change was observed in the peptide-evoked cell index in the presence of PMX53 (see Fig. 1D). The xCELLigence activation profiles are depicted in Fig. 2, where all the peptides even at a very high dose (100 µM) have a monophasic profile more similar to that of C3a than of C5a. Despite some being partial agonists at the C3a receptor, none of the peptides were able to antagonize C3a activation of HMDM when tested at 10 µM (data not shown).

### 3.4 Rat basophilic leukemia cell degranulation assay

**Fig. 3:**
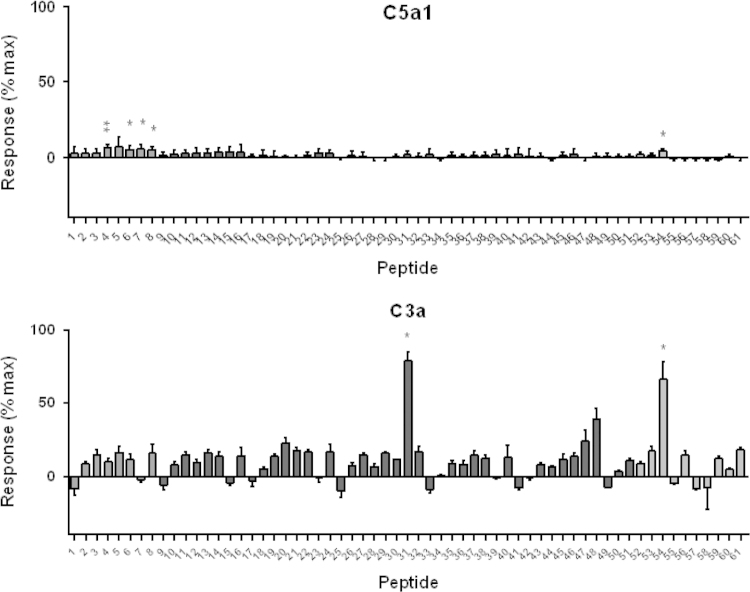
Agonist activity of peptides at human C5a_1_ and C3a receptors expressed in rat basophilic leukemia cells (RBL-2H3). Peptides were dissolved in DMSO and incubated at 50 μM with RBL-2H3 cells transfected with the appropriate receptor for 15 min. Degranulation was measured as the secretion of β-hexosaminidase. Results are expressed relative to maximal stimulation with 200 nM C5a (C5a_1_) or 100 nM hexapeptide agonist FLPLAR (C3a) after subtraction of background. Statistical significance of the difference from zero was assessed using a one-sample *t* test (^⁎^*P*<0.05; ^⁎⁎^*P*<0.001). Bars are shaded according to peptide length (see Supplementary Table 2).

RBL-2H3 cells transfected with either human C5a_1_ or human C3a receptor (Cain and Monk, 2002) were used to confirm hits detected in HMDM, using degranulation as a read-out (Monk et al., 1994). In cells transfected with C5a_1_, very weak agonist activity was detected in two groups of peptides, although even at 100 µM (the maximal dose achievable in the presence of the DMSO, which has adverse effects >1%), the activation obtained was 5–10% of the maximum activation achieved by a high dose of C5a (Fig. 3, top panel). In contrast, two peptides (31 and 54) produced very strong activation of C3a receptor (Fig. 3, lower panel) at 100 µM. In antagonist assays using two different doses of C5a that caused 50% or 100% degranulation, none of the peptides had any antagonist activity, even when pre-incubated with cells at the maximum achievable dose, 100 µM (data not shown).

## 4 Discussion

The design of C5a receptor agonists and antagonists employed only the structure of C5a. Thus we were only able to use the fold specificities as a metric for ranking the sequences. Four separate flexible templates were used for the design, combining them with eight runs defining the mutation sets and biological constraints for a total of 32 runs (see Supplementary Table 1). With all these combinations, we generated 6400 total sequences. We reduced this set to 61 sequences (Supplementary Table 2) by selecting the top few sequences from each run, or highly ranked sequences that looked interesting based on inspection (e.g. using different mutations not seen in the top ranked sequences). The majority of the runs reproduced the G73W mutation seen in the potent agonist by Finch et al. (1999).

These 61 sequences were then synthesized and screened using the xCELLigence, a label-free impedance-based biosensor, in human monocyte derived macrophages (HMDM), which have been shown to express the C5a_1_ receptor in relatively high abundance. The xCELLigence was our primary choice for testing as it is sensitive enough to allow detection in native cells and provides a pathway independent cell activation response, making it a useful broad screening tool (Halai and Cooper, 2012). From the 61 peptide set tested, five clear hits (20, 31, 47, 49 and 54) were identified (Fig. 1A).

Interestingly, the receptor signaling profile generated by these hits more closely resembled that of C3a than C5a_1_, as highlighted in Fig. 2. The receptor activation and kinetics between C3a and C5a_1_ differ significantly, where the evolution of the signal peaks at ~1 min after peptide addition for C3a receptor and ~4 min after peptide addition for C5a_1_. The intensity of the signal varies too, where a cell index of 1.98 and 4.53 is observed for C3a receptor and C5a_1_, respectively. As the receptors for both C3a and C5a are expressed in HMDM, it is entirely feasible these peptides were activating C3a receptor instead of C5a_1_. To discriminate whether the signal observed for peptides 20, 31, 47, 49 and 54 on HMDM was attributable to C3a or C5a, a C3a receptor antagonist, SB 290157, and C5a_1_ antagonist, PMX53, was employed.

C3a receptor antagonist SB 290157 showed a dose-dependent inhibition of the signal observed for peptides 20, 31, 47, 49 and 54 (Fig. 1C), alluding to C3a receptor being the target receptor for these hits. These observations were further supported, when no dose-dependent inhibition of the signal was observed in the presence of C5a_1_ antagonist, PMX53 (Fig. 2D), despite incubating at a higher starting concentration than SB 290157. To further validate this specificity, the peptides were tested in RBL-2H3 cells transfected with either human C5a_1_ or human C3a receptor. The peptides were screened as agonists against both C3a receptor and C5a_1_. As the results in Fig. 3 show, only C3a receptor responded strongly to the peptides, with high levels of degranulation produced by a subset of the peptides identified in the HMDM screen. The differences in the screens could be due to receptor expression levels (likely to be higher in the transfected cell line) or to the different read-out. Tested as C5a_1_ antagonists, the peptides had no effect at all confirming the very weak or absent interaction with this receptor.

Although these peptides were designed using the C-terminal domain of C5a, they demonstrated activity at C3a receptor, but not C5a_1_. C3a receptor/C5a_1_ promiscuity is not unusual and has been previously observed for whole C3a and a synthetic peptide agonist (Ames et al., 1996) and a chimeric C5a with the C3a C-terminal pentapeptide (Bautsch et al., 1992). In fact, early efforts to create potent and selective antagonists of C5a_1_ led to only partial agonists (Taylor and Fairlie, 2005). This may be due to the fact that most potent peptides had a mutation of Gly to Pro at position 73, disrupting the turn conformation of the C-terminus. Researchers at Merck were some of the first to succeed in creating a small peptide inhibitor of C5a receptors. Their compound, the hexapeptide MeFKPdChaFr (N-methylphenylalanine-Lys-Pro-d-cyclohexylalanine-Phe-d-arginine), was shown to be an antagonist but also had partial agonist behavior (Drapeau et al., 1993). Finch et al. (1999) optimized the Merck peptide using a cyclization strategy. Their best antagonist, AcF[OPdChaWR] (AcPhe[Lornithine-Pro-d-cyclohexylalanine-Trp-Arg], brackets denote cyclization; i.e. PMX53), had receptor affinity in the low nanomolar range (Taylor and Fairlie, 2005). Work by the same researchers (Finch et al., 1997, Kawatsu et al., 1996) also reported the competitive agonist YSFKDMPLaR (Tyr-Ser-Phe-Lys-Pro-Met-Pro-Leu-d-Ala-Arg), although this peptide too shows promiscuity for C3a receptor (Scully et al., 2010). In this study, despite applying our de novo design framework to find novel agonists and antagonists of C5a_1_, we identified novel sequences that agonize the C3a receptor.

Recent evidence (Wu et al., 2013) has shown agonism of the C3a receptor may have a protective role in acute intestinal injury. Wu et al. (2013) demonstrated the key role of C3a receptor in regulating neutrophil mobilization after acute intestinal injury, opening up C3a receptor agonism as a potential therapeutic avenue. Using the sequences identified in this study, new peptide mimetics can be designed and synthesized. Analysis of the sequences (Supplementary Table 1) of the active peptides reveals a conserved ‘Leucine (L)-Tryptophan (W)-Arginine (R)’ sequence at the C termini, as well as a conserved Tyrosine (Y) two amino acids before the LWR motif. The Leucine and the Arginine are also found in the same positions at the C termini in the superagonist ‘W-W-G-K-K-Y-R-A-S-K-L-G-L-A-R’ (Bellows-Peterson et al., 2012, Ember et al., 1991), and seem to be important for determining the activity towards C3a receptor. This is not surprising as Ember et al. (1991) described the C terminus interacting with the primary binding site and the N terminus with a secondary site. As we have already been able to show activity towards the C3a receptor, future work to increase potency should focus on the N termini, more specifically incorporating hydrophobic residues that are implicated to form interactions with the secondary site according to Ember et al. (1991). However, our other peptide sequences harboring these amino acids did not show any significant activity, so these amino acids may be working in conjunction with other key residues in the active three-dimensional structural conformation to elicit the activity observed.

Systematic and sequential amino acid replacements and truncations of the active peptide sequences may provide further insight into the key residues critical for the agonist activity observed at the C3a receptor. This knowledge will be key in optimizing the potencies of these peptides further, in the hope of developing them as therapeutics (Reid et al., 2013).

In conclusion, our screen identified several potent small peptide C3a receptor agonists. The lack of C5a_1_ hits, and hits for C3a receptor indicates promiscuity of C3a receptor and C5a_1_ ligand binding sites. Presumably the smaller size of these peptides enabled binding to C3a receptor. These C3a receptor agonist hits could be extended towards acute therapies to limit neutrophil mobilization in disease (Wu et al., 2013) or used as a basis for development of C3a receptor antagonists for chronic inflammatory disease.
