# Supplementary material for: Derivation of ligands for the complement C3a receptor from the C-terminus of C5a
Source: Eur J Pharmacol. 2014 Dec 15;745:176–81. doi: 10.1016/j.ejphar.2014.10.041 (PMC4263610; doi:10.1016/j.ejphar.2014.10.041)
Supplement: Supplementary file 1 — Supplementary material [file mmc1.docx]

**Supplementary data**

## Broad overview of Computational Design

A two-stage de novo protein design framework was applied in the design of the C5a-derived peptides ([Bellows-Peterson et al., 2012](#_ENREF_1); [Bellows et al., 2010a](#_ENREF_2); [Bellows et al., 2010b](#_ENREF_3); [Fung et al., 2008](#_ENREF_5); [Fung et al., 2005](#_ENREF_6); [Fung et al., 2007](#_ENREF_7); [Klepeis et al., 2003](#_ENREF_10); [Klepeis et al., 2004](#_ENREF_11)). The ﬁrst stage generates novel sequences by selecting mutations with favorable potential energies in a given ﬂexible design template. In addition to this ﬂexible design template, the specification of allowed mutation constraints, force ﬁeld, and biological constraints are also used as input to the optimization model. The second stage computationally validates these sequences by either fold speciﬁcity ([Fung et al., 2008](#_ENREF_5); [Fung et al., 2007](#_ENREF_7)) or approximate binding afﬁnity([Bellows et al., 2010a](#_ENREF_2); [Bellows et al., 2010b](#_ENREF_3)). Fold speciﬁcity is a measure of how well the sequence adopts the template fold, while approximate binding afﬁnity is a measure of how well the sequence binds to a target protein. Since information about C5a binding to its receptor was unavailable for this case, only fold speciﬁcity was used to validate the designed sequences. Two different ways of calculating fold speciﬁcity were utilized, the original method described by Fung *et al*. ([Fung et al., 2008](#_ENREF_5)) that uses the AMBER force ﬁeld ([Cornell et al., 1995](#_ENREF_4)) and a new method that uses the FAMBE-pH force field ([Vorobjev et al., 2008](#_ENREF_16)).

Four flexible design templates were used. The ﬁrst template consisted of 20 NMR solution structures ([Zhang et al., 1997](#_ENREF_17)), and the remaining three consisted of 20-ns molecular dynamics (MD) simulation trajectories. All of the MD simulations started with 1000 steps of energy minimization, but differed in the equilibration steps before the production run. The MD non-thermal simulation had no further equilibration following minimization. All atoms went from being at rest to having velocities cor­responding to 300 K immediately. The MD thermal simulation underwent heat equilibration in which the temperature of the system was gradually raised from 0 to 300 K. The MD constrained equilibration simulation utilized harmonic constraints to allow the water to ﬁrst equilibrate, while constraining the dynamics of the protein. The constraints were then gradually reduced to let the protein free. The MD templates showed large variability in the C-terminus, which is involved in binding to the receptor and is the basis of our design. The MD simulations were performed using the program NAMD ([Phillips et al., 2005](#_ENREF_13)). These flexible templates were used as input in the distance bin sequence selection model.

Supplementary Figure 1 shows an overlay of the NMR ﬂexible template and a representative structure from each of the MD design templates. In general, all the templates consisted of ﬁve α-helices. In the NMR template (Fig. 1A), the C-terminal helix formed contacts with the N-terminal helix, almost locking them together. For the MD templates (Figs. 1B-D), the ﬁrst MD snapshot corresponded to the structure seen in Fig. 1A, that is the N-terminus and C-terminus were in a “closed” con­formation. As the runs progressed, however, the thermal and non-thermal runs lost helicity in the C-terminus (Figs. 1B and C). In addition, the C-terminus showed great ﬂexibility in all three MD templates, opening to the right in the thermal and constrained equilibration runs (Figs. 1B and D) and to the left in the non-thermal run (Fig. 1C). Because of these differences, and the importance of the C-terminus in binding to the receptor, we performed each of our design runs four times, one for each of the templates.

In addition to the flexible template, several sets of mutation constraints were used for the design of octa-, hepta-, and hexa-peptides based on the structure of the C-terminus of C5a. The use of the structure of the C5a terminus was based on the knowledge that the C-terminus is involved in binding to its receptors. Two mutation sets were constructed for octapeptides (MS1-MS2), five mutation sets for heptapeptides (MS3-MS7), and one mutation set for hexapeptides (MS8). These mutation sets are provided in Table 1 and detail the constraints used in all 32 design runs performed in this study. In addition to the mutation constraints, homologous protein statistics were derived from a blast run in order to calculate appropriate upper and lower bounds on the frequency of each amino acid and the overall charge. From these statistics a variety of charge constraints were included in the runs as follows: Sets MS1-MS2 limited the charge to +1-+2, sets MS3-MS5 fixed the charge to +1, sets MS6-MS7 fixed the charge to +2, and MS8 fixed the charge to +2.

Since a flexible design template was available, the distance bin sequence selection model was used to generate low-energy sequences that are favorable in the design template. The distance bin model is formulated as an integer linear optimization model (ILP) and was implemented in this application as it was originally proposed by Fung et al. ([Fung et al., 2008](#_ENREF_5)). All runs used the centroid-centroid 8 bin forcefield detailed in Rajgaria *et al.* ([Rajgaria et al., 2008](#_ENREF_15)).

The second stage validates the sequences from stage one using a fold specificity metric. Fung et al. ([Fung et al., 2008](#_ENREF_5)) proposed this metric which generates an ensemble of structures using torsional angle dynamics through CYANA 2.1 ([Guntert, 2004](#_ENREF_8); [Guntert et al., 1997](#_ENREF_9)), followed by an energy minimization in TINKER ([Ponder, 1998](#_ENREF_14)) using the full-atomistic AMBER force field ([Cornell et al., 1995](#_ENREF_4)). Here we also present an improvement on the fold specificity method, which uses the FAMBE-pH force field ([Vorobjev et al., 2008](#_ENREF_16)) instead of AMBER to calculate the energies of the conformers.

The fold specificity for a given mutant sequence using either the AMBER or FAMBE-pH force field is calculated by Equation 1:

$f_{spec}=\frac{\sum_{i\in Novel} e^{-\beta E_{i}}}{\sum_{j\in Native} e^{-\beta E_{j}}}$ (1)

Where $\beta=\frac{1}{k_{B}T}$, $E_{i}$ is the AMBER or FAMBE-pH energy of the $i$th conformer of the protein ensemble generated for a particular designed sequence, and $E_{j}$ is the AMBER of FAMBE-pH energy of the $j$th conformer of the protein ensemble generated for the native sequence.

**Supplementary Table 1. Mutation constraints used as input into the sequence selection stage**. SASA designation for the four C5a design templates are given as follows: B indicates hydrophobic amino acids (A, I, L, M, F, W, Y, V), Z indicates hydrophilic amino acids (R, N, D, Q, E, G, H, K, P, S, T), and O indicates all amino acids except Cys were allowed at that position. A “+” indicates that only positively charged mutations (R and K) were allowed in that position. A “+/-” indicated that only charged mutations (R, K, E, D) were allowed in that position. An “N/A” indicates that this position was not included in the run. The native residue was also always allowed at each mutable position. Sequence numbering is with respect to the full C5a sequence.

|  | |  | C5a C-terminal Residue Position | | | | | | | |  |
| --- | --- | --- | --- | --- | --- | --- | --- | --- | --- | --- | --- |
| Type | Set | Template | 67 | 68 | 69 | 70 | 71 | 72 | 73 | 74 | Run # |
| Octa- | MS1 | NMR/MDt | O | Z | Z | O | Z | O | B | R | 1/2 |
|  |  | MDnt | O | Z | O | Z | Z | Z | B | R | 3 |
|  |  | MDc | O | Z | O | O | Z | Z | O | R | 4 |
|  | MS2 | NMR/MDt | B | Z | Z | O | Z | O | B | R | 5/6 |
|  |  | MDnt | B | Z | O | Z | Z | Z | B | R | 7 |
|  |  | MDc | B | Z | O | O | Z | Z | O | R | 8 |
| Hepta- | MS3 | NMR/MDt | N/A | Z | Z | O | Z | O | B | R | 9/10 |
|  |  | MDnt | N/A | Z | O | Z | Z | Z | B | R | 11 |
|  |  | MDc | N/A | Z | O | O | Z | Z | O | R | 12 |
|  | MS4 | NMR/MDt | N/A | + | Z | O | Z | O | B | R | 13/14 |
|  |  | MDnt | N/A | + | O | Z | Z | Z | B | R | 15 |
|  |  | MDc | N/A | + | O | O | Z | Z | O | R | 16 |
|  | MS5 | NMR/MDt | N/A | +/- | Z | O | Z | O | B | R | 17/18 |
|  |  | MDnt | N/A | +/- | O | Z | Z | Z | B | R | 19 |
|  |  | MDc | N/A | +/- | O | O | Z | Z | O | R | 20 |
|  | MS6 | NMR/MDt | N/A | + | Z | O | Z | O | W | R | 21/22 |
|  |  | MDnt | N/A | + | O | Z | Z | Z | W | R | 23 |
|  |  | MDc | N/A | + | O | O | Z | Z | W | R | 24 |
|  | MS7 | NMR/MDt | N/A | +/- | Z | O | Z | O | W | R | 25/26 |
|  |  | MDnt | N/A | +/- | O | Z | Z | Z | W | R | 27 |
|  |  | MDc | N/A | +/- | O | O | Z | Z | W | R | 28 |
| Hexa- | MS8 | NMR/MDt | N/A | N/A | Z | O | Z | O | W | R | 29/30 |
|  |  | MDnt | N/A | N/A | O | Z | Z | Z | W | R | 31 |
|  |  | MDc | N/A | N/A | O | O | Z | Z | W | R | 32 |


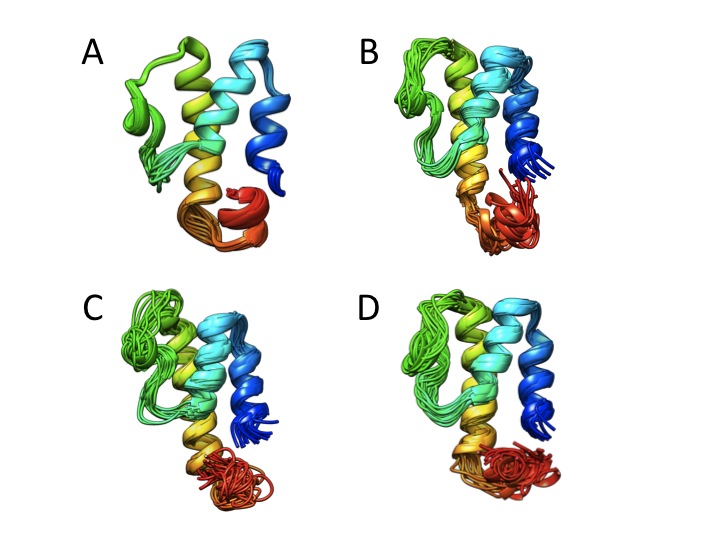


**Supplementary Figure 1.** (a) Overlay of 20 NMR solution structures of C5a, PDB code: 1KJS ([Zhang et al., 1997](#_ENREF_17)). (b) Overlay of 20 MD snapshots corresponding to the thermal run. (c) Overlay of 20 MD snapshots corresponding to the non-thermal run. (d) Overlay of 20 MD snapshots corresponding to the constrained equilibration run. For all ﬁgures, N-terminus is blue and C-terminus is red. Molecular graphics images were produced using the UCSF Chimera package from the Computer Graphics Laboratory, University of California, San Francisco ([Pettersen et al., 2004](#_ENREF_12)).

**Supplementary Table 2** Computationally predicted peptide sequences that were experimentally tested. The peptide numbers correspond to those of Figure 2a. Bold-faced characters denote those sequences that were found to have agonist activity (see Table 3). A * next to a Peptide # indicated sequences chosen using the new FAMBE-pH fold specificity.

| **Peptide #** | **Sequence** | **Molecular Weight** | **Net Charge** |  | **Peptide #** | **Sequence** | **Molecular Weight** | **Net Charge** |
| --- | --- | --- | --- | --- | --- | --- | --- | --- |
| Native | HKDMQLGR |  | 1 |  | ***31** | **Ac-RHYPLWR** | **1069.22** | **2** |
| 1 | Ac-FDNYKFWR | 1217.34 | 0 |  | *32 | Ac-RHYPYWR | 1119.24 | 2 |
| 2 | Ac-MQQWQVFR | 1164.34 | 0 |  | 33 | Ac-RLYDRWR | 1106.24 | 1 |
| 3 | Ac-MQQWRVFR | 1192.4 | 1 |  | 34 | Ac-RLYNNWR | 1063.17 | 1 |
| 4 | Ac-NDWNNRLR | 1129.19 | 0 |  | 35 | Ac-RLYREWR | 1120.27 | 1 |
| 5 | Ac-NDWNNRQR | 1144.16 | 0 |  | 36 | Ac-RNLNQWR | 1028.13 | 1 |
| 6 | Ac-NDWNNRYR | 1179.21 | 0 |  | 37 | Ac-RNQNNWR | 1029.07 | 1 |
| 7 | Ac-NDWNQRQR | 1158.19 | 0 |  | 38 | Ac-RNYDYWR | 1114.18 | 0 |
| 8 | Ac-NNNWQQQR | 1129.15 | 0 |  | 39 | Ac-RQNNNWR | 1029.07 | 1 |
| 9 | Ac-DRYERWR | 1122.2 | 0 |  | 40 | Ac-RWNDNQR | 1030.06 | 0 |
| 10 | Ac-DWNNRYR | 1065.1 | 0 |  | 41 | Ac-RWNNDQR | 1030.06 | 0 |
| 11 | Ac-EDYRRWR | 1122.2 | 0 |  | *42 | Ac-RWIHHGR | 1003.13 | 3 |
| 12 | Ac-EEYRRWR | 1136.23 | 0 |  | *43 | Ac-RWHHHGR | 1027.11 | 4 |
| 13 | Ac-ERYDRWR | 1122.2 | 0 |  | *44 | Ac-RLYERWR | 1120.27 | 1 |
| *14 | Ac-HHIRYWR | 1109.25 | 3 |  | *45 | Ac-RLLHHWR | 1059.23 | 3 |
| *15 | Ac-HHLRFWR | 1093.25 | 3 |  | *46 | Ac-RLYPHWR | 1069.22 | 2 |
| *16 | Ac-HHLRYWR | 1109.25 | 3 |  | ***47** | **Ac-RLYPLWR** | **1045.24** | **1** |
| *17 | Ac-HRYNPWR | 1070.17 | 2 |  | *48 | Ac-TLLRLWR | 999.21 | 1 |
| 18 | Ac-NNLNQWR | 986.05 | 0 |  | ***49** | **Ac-TIYRLWR** | **1049.23** | **1** |
| 19 | Ac-NNLNRWR | 1014.1 | 1 |  | *50 | Ac-TFYNKWR | 1056.18 | 1 |
| **20** | **Ac-NNYNLWR** | **1021.09** | **0** |  | *51 | Ac-TFYRLWR | 1083.25 | 1 |
| 21 | Ac-NNYNQWR | 1036.06 | 0 |  | *52 | Ac-RYPYWR | 982.1 | 1 |
| 22 | Ac-NWNNQQR | 1001.02 | 0 |  | *53 | Ac-HKPYWR | 928.05 | 2 |
| 23 | Ac-QNNNRWR | 1029.07 | 1 |  | ***54** | **Ac-RYPLWR** | **932.08** | **1** |
| 24 | Ac-QWNNNQR | 1001.02 | 0 |  | *55 | Ac-RYPHWR | 956.07 | 2 |
| 25 | Ac-RDLNNWR | 1015.09 | 0 |  | *56 | Ac-LLNRWR | 899.06 | 1 |
| 26 | Ac-RDNNNWR | 1016.03 | 0 |  | *57 | Ac-LLRNWR | 899.06 | 1 |
| 27 | Ac-RDNNQWR | 1030.06 | 0 |  | *58 | Ac-FLRLWR | 932.13 | 1 |
| 28 | Ac-RDYNLWR | 1064.16 | 0 |  | *59 | Ac-LIRLWR | 898.11 | 1 |
| *29 | Ac-RHLHHWR | 1083.21 | 4 |  | *60 | Ac-LIPKWR | 854.05 | 1 |
| *30 | Ac-RHYPHWR | 1093.21 | 3 |  | *61 | Ac-LLPKWR | 854.05 | 1 |

**References**

Bellows-Peterson, M.L., Fung, H.K., Floudas, C.A., Kieslich, C.A., Zhang, L., Morikis, D., Wareham, K.J., Monk, P.N., Hawksworth, O.A., Woodruff, T.M., 2012. De Novo Peptide Design with C3a Receptor Agonist and Antagonist Activities: Theoretical Predictions and Experimental Validation. Journal of Medicinal Chemistry 55, 4159-4168.

Bellows, M.L., Fung, H.K., Taylor, M.S., Floudas, C.A., Lopez de Victoria, A., Morikis, D., 2010a. New compstatin variants through two de novo protein design frameworks. Biophys J 98, 2337-2346.

Bellows, M.L., Taylor, M.S., Cole, P.A., Shen, L., Siliciano, R.F., Fung, H.K., Floudas, C.A., 2010b. Discovery of entry inhibitors for HIV-1 via a new de novo protein design framework. Biophys J 99, 3445-3453.

Cornell, W.D., Cieplak, P., Bayly, C.I., Gould, I.R., Merz, K.M., Ferguson, D.M., Spellmeyer, D.C., Fox, T., Caldwell, J.W., Kollman, P.A., 1995. A 2nd Generation Force-Field For The Simulation Of Proteins, Nucleic-Acids, And Organic-Molecules. J Am Chem Soc 117, 5179-5197.

Fung, H.K., Floudas, C.A., Taylor, M.S., Zhang, L., Morikis, D., 2008. Toward Full-Sequence De Novo Protein Design with Flexible Templates for Human Beta-Defensin-2. Biophys J 94, 584-599.

Fung, H.K., Rao, S., Floudas, C.A., Prokopyev, O., Pardalos, P.M., Rendl, F., 2005. Computational Comparison Studies of Quadratic Assignment Like Formulations for the In Silico Sequence Selection Problem in De Novo Protein Design. J Comb Optim 10, 41-60.

Fung, H.K., Taylor, M.S., Floudas, C.A., 2007. Novel Formulations for the Sequence Selection Problem in De Novo Protein Design with Flexible Templates. Optim Method Softw 22, 51-71.

Guntert, P., 2004. Automated NMR structure calculation with CYANA. Methods in molecular biology 278, 353-378.

Guntert, P., Mumenthaler, C., Wuthrich, K., 1997. Torsion angle dynamics for NMR structure calculation with the new program DYANA. Journal of molecular biology 273, 283-298.

Klepeis, J.L., Floudas, C.A., Morikis, D., Tsokos, C.G., Argyropoulos, E., Spruce, L., Lambris, J.D., 2003. Integrated Structural, Computational and Experimental Approach for Lead Optimization: Design of Compstatin Variants with Improved Activity. J Am Chem Soc 125, 8422-8423.

Klepeis, J.L., Floudas, C.A., Morikis, D., Tsokos, C.G., Lambris, J.D., 2004. Design of Peptide Analogs with Improved Activity using a Novel de novo Protein Design Approach. Ind Eng Chem Res 43, 3817-3826.

Pettersen, E.F., Goddard, T.D., Huang, C.C., Couch, G.S., Greenblatt, D.M., Meng, E.C., Ferrin, T.E., 2004. UCSF Chimera--a visualization system for exploratory research and analysis. Journal of computational chemistry 25, 1605-1612.

Phillips, J.C., Braun, R., Wang, W., Gumbart, J., Tajkhorshid, E., Villa, E., Chipot, C., Skeel, R.D., Kale, L., Schulten, K., 2005. Scalable molecular dynamics with NAMD. Journal of computational chemistry 26, 1781-1802.

Ponder, J.W., 1998. TINKER, software tools for molecular design. 1998.

Rajgaria, R., McAllister, S.R., Floudas, C.A., 2008. Distance Dependent Centroid to Centroid Force Fields Using High Resolution Decoys. Proteins 70, 950-970.

Vorobjev, Y.N., Vila, J.A., Scheraga, H.A., 2008. FAMBE-pH: A Fast and Accurate Method to Compute the Total Solvation Free Energies of Proteins. J Phys Chem B 112, 11122-11136.

Zhang, X.L., Boyar, W., Toth, M.J., Wennogle, L., Gonnella, N.C., 1997. Structural Definition of the C5a C terminus by Two-dimensional Nuclear Magnetic Resonance Spectroscopy. Proteins: Structure, Function, and Bioinformatics 28, 261-267.
